# Protective effects of resveratrol and naringenin against nonylphenol-induced oxidative stress in rats

**DOI:** 10.1186/s13568-024-01788-z

**Published:** 2025-01-08

**Authors:** Haitham S. Elewa, Dawlat A. Salama, Mohamed S. Hikal, Mohamed F. Abd El hamid, Mohamed H. Eid, Fatma M. A. Khalil, Muayad S. Albadrani, Khaled Abdelaal, Ahmed I. El-Tokhy

**Affiliations:** 1 Biochemistry Department, Faculty of Agriculture, Ain Shams University, Cairo, Egypt; 2 Institute of Environmental Management, Faculty of Earth Science, University of Miskolc, Miskolc- Egyetemváros, 3515 Hungary; 3 Geology Department, Faculty of Science, Beni-Suef University, Beni-Suef, 65211 Egypt; 4 Applied College, Unit of health specialties, basic sciences and their applications, King Khalid University, Mohayil, Abha, Asir 61421 Saudi Arabia; 5 Department of Family and Community Medicine and Medical Education, College of Medicine, Taibah University, Al-Madinah Al-Munawara, 42353 Saudi Arabia; 6 Plant Pathology and Biotechnology Lab, Faculty of Agriculture, EPCRS Excellence Center, Kafrelsheikh University, Kafr el-Sheikh, 33516 Egypt; 7 Plant Protection Department, Faculty of Agriculture, Beni-Suef University, Beni-Suef, 62511 Egypt

**Keywords:** Xenoestrogen, Resveratrol, Naringenin, Estrogen receptor, Endocrine disruption

## Abstract

Nonylphenol (NP) is a ubiquitous environmental endocrine disrupting chemical and oxidative stress inducer in biological systems. Resveratrol (RES) and Naringenin (NG) are phytochemicals possessing antioxidant properties and estrogenic activity. This study was conducted to investigate the toxicity of NP and the mitigating effects of RES and NG on NP toxicity in rats. Thirty male rats were classified into 5 groups as follows: 1- Normal control (NC), 2- Dimethyl sulfoxide (DMSO) group, 3- NP group, 4- NP + RES and 5- NP + NG. Results revealed that NP treatment significantly decreased the activities of superoxide dismutase, Catalase, Glutathione peroxidase and Glutathione content in blood, liver and kidney compared to NC and DMSO groups. Conversely, activity of Glutathione-s-transferase was significantly elevated in blood and decreased in liver and kidney. Moreover, significant escalation was observed in the levels of Malondialdehyde. Also, NP treatment led to a significant decrease in serum total testosterone and testis weight, accompanied with concurrent elevation in estradiol level compared to NC and DMSO groups. All the recorded effects induced by NP treatment were effectively countered by co-treatment with RES or NG. In addition, molecular docking studies were carried out to reveal the interactions between NP, RES, NG and estrogen receptor beta which provide a possible mechanism for their potential estrogenic activity. Overall, our study gives a deeper understanding of the toxic effect of NP on antioxidant capacity and endocrine functions as well as the potential therapeutic utility of RES and NG in alleviating these adverse effects.

## Introduction

Humans and living organisms such as plants and aquatic species are exposed to many harmful chemical compounds that are present in the environment. These compounds are abiotic stress factors such as salinity and drought and may cause oxidative stress (Abdou et al. 2023; Alshammari et al. 2024a, b). Some of these compounds show their harmful effect quickly, while others show their effect after chronic and long-term exposure to them, Nonylphenol (NP) is one of these compounds. It is a synthetic lipophilic xenoestrogen that consists of a phenol ring bearing a side chain of 9 carbon atoms. NP is a precursor of the important non-ionic surfactants NP ethoxylates (NPEOs), which are used as adjuvant in herbicides and pesticide formulations in industry, agriculture, food, detergents, paints, personal care products, plastics, cosmetics, emulsifiers, resins, wetting and dispersing agents, lubricants, polystyrene tubes, paper, textile, and household sectors. NP also reacted to form tris(4-nonyl-phenyl) phosphite (TNPP) an antioxidant used as a stabilizer in plastic food packaging, although it does contain residual NP. Barium and calcium salts of NP are used as heat stabilizers for poly vinyl chloride (PVC) (Bhandari et al. 2021; Iqbal and Bhatti 2015; Cheng et al. 2014).

The occurrence of NP has been reported in different environmental compartments worldwide, as well as within humans and other biota (Diao et al. 2017; Lin et al. 2017; Peng et al. 2017). It is released and accumulated in the environment, commonly found in soil, water and air, and can also enter the food chain from the polluted environment. So, NP contamination has been found both in foods (including fish, meat and vegetables) and the plastics used in food processing and packing (Gyllenhammar et al. 2012; Soares et al. 2008). Therefore, Human exposure to NP occurs through its use in pesticides, plastic food packaging, and its presence in household products such as detergents and cosmetics, human beings are inevitably exposed to NP via ingestion, inhalation and dermal routes throughout their whole lifetimes, with the food intake as a major route via the food chain from bioaccumulation in the polluted environment (Gyllenhammar et al. 2012; Balakrishnan et al. 2011; Nappi et al. 2016). It has gained medical attention due to its potential negative effects and adverse consequences on human health and the environment. Numerous health issues of NP result from two routes: 1- It leads to oxidative stress via generation of reactive oxygen species (ROS), that leads to the imbalance of oxidants and antioxidants and increase lipid peroxidation (Ijaz et al. 2021). Increase of the oxidative stress, leading to the formation of hydroxyl radical and the decrease of antioxidant capacity (Mao et al. 2011). 2- It causes an imbalance and disturbance in the hormonal system of the endocrine glands, as NP falls within a group of compounds known as Synthetic Xenoestrogens, it is a group of chemically synthesized compounds that are somewhat similar to the estrogen hormone in structure and mimic its function, as it has the ability to bind to estrogen receptors, but to varying degrees. NP is well recognized for its ability to disrupt endocrine properties owing to its similarity to estrogen. NP can act on different molecular targets and generate multiple toxicities in body such as developmental defects, reproductive diseases, liver and kidney impairments and carcinogenic effects (Huang et al. 2019; Di et al. 2018; Ho and Watanabe 2018; Rattan et al. 2017).

We designed this study to examine the toxic effects of NP on the levels of systemic and organ-specific antioxidant enzymes, with a particular emphasis on glutathione-S-transferase (GST), catalase (CAT), superoxide dismutase (SOD), and glutathione peroxidase (GPx). These enzymes are known for their ability to neutralize ROS and preserve redox equilibrium within cells and tissues, thus combating oxidative stress and lipid peroxidation, as indicated by malondialdehyde (MDA) levels. Additionally, we investigated the possible protective effects against NP-induced oxidative damage offered by phytochemical substances such as NG (NG) and RES (RES). NG and RES, both well characterized antioxidants, were tested with NP to evaluate their ability to alleviate NP induced oxidative stress and restore antioxidant enzymes level (Ray et al. 2020; Kourouma et al. 2015). We extended our investigations to study NP induced endocrine-disturbing effects, with a focus on how it affected testis weight, testosterone and estradiol levels, all are indicators of disturbed reproductive and hormonal balance. We employed gene enrichment studies, protein-protein interaction and molecular docking analysis to gain better insights into the molecular mechanisms behind NP-induced oxidative stress and the possible protective effects of NG and RES. In addition, molecular docking studies were carried out to reveal the interactions between NP, RES, NG and estrogen receptor beta which provide a possible mechanism for their potential estrogenic activity. Overall, our work underscores the intricate impacts of NP exposure on oxidative stress, antioxidant defence mechanisms, organ function, and endocrine disruption and the significance of taking proactive measures to reduce NP-related health concerns.

## Materials and methods

### Chemicals

All chemicals used in this study were analytical grade and obtained from Sigma-Aldrich Chemical Co. Natural sources of RES are grapes (*Vitis vinifera)*, blueberries (*Vaccinium corymbosum*), peanut (*Arachis hypogaea*) and pistachio nut (*Pistacia vera*).

Natural sources of NG are grapefruit (Citrus paradisi), sweet orange (Citrus sinensis), and tomato (*Lycopersicon esculentum*) and rosemary (*Rosmarinus officinalis*).

### Biochemical parameters kits

All kits used in this study were purchased from biodiagnostic Company. Cairo, Egypt.

### Animals

Thirty adult male albino rats of similar weights and ages about (120 g) were chosen as an animal model for the present study. Rats were obtained from Animal Health Institute, Dokki, Giza, Egypt. Rats were maintained on a balanced standard diet and water ad-libitum. Animals were kept for one week as an adaptation period before starting the experiment.

### Experimental design

After a housing-acclimatization period of one week as previously mentioned, Rats were randomly divided into 5 groups (6 rats per group) with average weight (150 ± 5 g). Experimental groups were as follows:

Group 1(NC), Normal Control group: only fed on normal diet.

Group 2 (DMSO), animals were treated only with Dimethyl sulfoxide (DMSO) and served as DMSO control group. DMSO used as a vehicle for NP, RES and NG.

Group 3 (NP), Nonylphenol (NP) Group: animals were treated with NP.

Group 4 (NP + RES), NP and Resveratrol (RES) Group: animals were treated with NP and RES.

Group 5 (NP + NG), NP and Naringenin (NG) group: animals were treated with NP and NG.

### Administration and doses of NP and phytochemicals

NP was administered to rats orally by gavage syringe in a dose of 5 mg/kg body weight. RES and NG were administered to rats orally by gavage syringe in a dose of 10 mg/kg body weight. The doses of NG and RES were selected based on previously reported protective and antioxidant properties of this compound in rats (Butchi et al. 2011; Roy et al. 2013). NP, RES, NG and DMSO were gavaged daily from first day to the end of the experiment (90 days). DMSO is used as a vehicle for NP, RES and NG, so a group for DMSO control was designed. RES and NG treatments were gavaged to rats before NP.

#### Preparation of serum, plasma and tissues homogenate

Blood samples were collected from rats by sacrificing at the end of the experiment. Rats were anaesthetized in an anesthetic box fumed with ether vapor, then animals were sacrificed by cervical decapitation. Blood was collected in non-heparinized and heparinized tubes. Non-heparinized blood was allowed to clot and centrifuged for serum separation. For plasma, the tubes contained heparin as anticoagulant. After 10 min of centrifugation at 3000 rpm, plasma and buffy coat were separated. The isolated red cells were washed three times with physiological saline (0.9%). Plasma and serum were tightly kept in sealed Eppendorf tubes at -18ºC until it was processed for biochemical assays. The tissues (liver and kidneys) were dissected out and washed using ice cold saline solution. A known amount of tissue was weighed and homogenized in an appropriate buffer for the estimation of various biochemical parameters.

### Biochemical assays

MDA level was estimated to measure the lipid peroxidation in accordance with a method stated by Buege and Aust (1978). CAT activity was determined by the protocol of Sinha (1972). SOD activity was evaluated using a technique presented by Nishikimi et al. (1972). Glutathione peroxidase (GPx) activity was measured by the method of Rotruck et al. (1973). Glutathione S-transferase (GST) activity was analyzed by a protocol given by Habig et al. (1974).

### Hormones assay

Serum testosterone (T) and estradiol (E2) were assayed by Competitive Chemiluminescent Immunoassay using automated instrument ADVIA Centaur, Royal Lab, Egypt. The testosterone was estimated using ADVIA Centaur TSTO kit and estrogen was estimated using ADVIA Centaur using E2-6 kit.

#### Testes weight

At the end of the experimental period, rats were anaethetized with light ether and the testes of each rat were removed and weighed.

#### Molecular docking

##### Ligand preparation

Ligand structure was drawn using Chem Draw 21.0.0 (PerkinElmer, Waltham, MA, USA) and saved as SDF files. Ligand energy was minimized by MM2 calculation, logP was calculated, and the structures of the ligands were converted to pdb file format using Chem3D 21.0.0. Nonpolar hydrogen atoms were deleted, Gasteiger charges were calculated, torsion root was detected, and the structures were saved as pdbqt file format using AutoDockTools-1.5.6.

#### Target protein preparation

The target protein, estrogen receptor beta (ERB) structure encoding 5TOA (Souza et al. 2017) was downloaded from Protein Data Bank “www.rcsb.org/. (accessed on 13, 20 November 2023)”. The target protein structure was prepared by deleting water and solvent molecules and ligands, adding polar hydrogen atoms, calculating Kollman charges, and saving the structure of target protein as pdbqt using AutoDockTools-1.5.6 (The Scripps Research Institute, San Diego, CA, USA).

#### Molecular docking procedures

The molecular interactions between the ligand binding domain of Estrogen receptor beta and Estrogen or NP or RES or NG, were determined using AutoDock Vina 1.2.0 (The Scripps Research Institute, San Diego, CA, USA**)** (Eberhardt et al. 2021). A random seed number was used and the exhaustiveness function was 8. A 21 × 31 × 43 Å grid box with 76 × 90 × 64 grid point spacing of 0.37 Å for ERB, was used for docking the ligand into the receptor binding domain.

#### Analysis and visualization of protein–ligand interactions

The conformers of each ligand were separated using vina_split command. The conformer with the highest affinity and in silico interactions between the ligands and ERB or ERA was analyzed and visualized using Discovery Studio-21 software (Dassault Systems BIOVIA San Diego, CA, USA).

#### Statistical analysis

All results calculated were presented as means ± SD from six replicates and subjected to one way analysis of ANOVA test. The means of different treatments were compared using Duncan’s multiple range test (L.S.D) at *p* ≤ 0.05. Statistical analyses were performed using SPSS statistical software (IBM SPSS Statistics, version 20) (Snedecor and Chochran 1980).

## Results

### Toxic effects of NP on systemic, liver and kidney SOD levels

To evaluate the toxic effect of NP on antioxidant capacity of the body, we measured superoxide dismutase (SOD) activity in erythrocytes, liver and kidney (Table 1) of rats treated with NP compared to either untreated rats (NC group) or rats treated only with the vehicle (DMSO). We observed a significant reduction in serum SOD activity following NP administration compared to the other two groups (1.13 ± 0.04 in NP group vs. 1.93 ± 0.02 in NC group and 1.92 ± 0.05 in DMSO group); (*P* < 0.05). To investigate whether we could ameliorate this effect using phytochemical compounds such as NG (NG) and RES (RES). We used them in dose 10 mg/Kg B.W, following co-administration of either RES or NG with NP, we observed a significant restoration of SOD activity compared to the NP-treated group (1.99 ± 0.04 in RES group and 1.89 ± 0.03 in NG group; (*P* < 0.05)). The erythrocyte SOD measurements were followed by SOD levels analysis in liver and kidney tissues, with consistent observations throughout, where about 41% reduction was observed in SOD levels measured in liver and kidney tissues of rats treated with NP compared to untreated rats or rats treated only with (DMSO). The levels were restored to nearly 100% compared to the normal control groups upon the co-administration of either RES or NG as shown in Table 1.

**Table 1:** Effect of NP exposure on blood, liver and kidney SOD in rats treated with RES or NG

Parameter	NC	DMSO	NP	NP + RES	NP + NG
Erythrocytes SOD (U/mg Hb)	1.93 ± 0.02	1.92 ± 0.05	1.13* ± 0.04	1.99 ± 0.04	1.89 ± 0.03
liver SOD (U/mg protein)	32.05 ± 0.35	31.99 ± 0.9	18.85* ± 1.66	33.3 ± 1.26	31.53 ± 1.98
Kidney SOD (U/mg protein)	21.28 ± 0.52	20.91 ± 0.84	12.49* ± 0.81	21.82 ± 1.01	20.56 ± 0.72

### Impact of NP exposure on systemic, liver and kidney catalase levels

We examined the systemic impact of NP on catalase, a remarkable antioxidant (Glorieux and Calderon 2017), by measuring its level in the serum samples. Following exposure to NP, a significant reduction in catalase levels was noted (from 421.68 ± 3.07 in NC group and 418.93 ± 4.3 in DMSO group to 326.77 ± 4.09; (*P* < 0.05) in the serum levels of NP treated group, indicating a systemic response to NP-induced oxidative stress.

To investigate the tissue-specific effects of NP, we evaluated catalase levels in liver and kidney tissues (Table 2). In both organs, NP exposure resulted in a notable decrease in catalase levels in both the liver (from 66.57 ± 1.71 and 66.14 ± 1.57 to 51.79 ± 2.69; (*P* < 0.05) and the kidney (from 37.18 ± 0.83 and 36.29 ± 1.26 to 25.54 ± 1.98; (*P* < 0.05). Selective restoration of liver and kidney catalase levels by RES co-administration with NP was reported. Meanwhile NG co-administration effectively restored systemic, liver, and kidney catalase levels as displayed in Table 2. The exhibited levels were comparable to those observed in control or DMSO-treated rats.

**Table 2:** Effect of NP exposure on blood, liver and kidney CAT in rats treated with RES or NG

Parameter	NC	DMSO	NP	NP + RES	NP + NG
CAT (U/ml blood)	421.68 ± 3.07	418.93 ± 4.3	326.77* ± 4.09	401.72* ± 3.12	412.25 ± 5.25
liver CAT (μmol H_2_O_2_/min/mg protein)	66.57 ± 1.71	66.14 ± 1.57	51.79* ± 2.69	64.93 ± 2.03	67.08 ± 2.81
kidney CAT (μmol H_2_O_2_/min/mg protein)	37.18 ± 0.83	36.29 ± 1.26	25.54* ± 1.98	36.12 ± 1.15	37.57 ± 1.14

#### Effect of NP on glutathione peroxidase (GPx) enzyme in blood, liver and kidney of the experimental rats

Significant reductions in glutathione peroxidase levels were observed in the blood, liver, and kidney tissues of the rats exposed to NP compared to both control and DMSO-treated groups (Table 3). Specifically, reductions of 46.68%, 27.35%, and 31.33% were observed in the blood, liver, and kidney tissues, respectively, compared to both control and DMSO-treated groups. Subsequent co-administration with either RES or NG resulted in normalization of glutathione peroxidase levels, apart from glutathione peroxidase levels in the kidney of the NP-treated group treated with RES, albeit they exhibited elevated trend as reported in Table 3.

**Table 3:** Effect of NP exposure on blood, liver and kidney GPx in rats treated with RES or NG

Parameter	NC	DMSO	NP	NP + RES	NP + NG
GPx (U/ml blood)	22.02 ± 0.76	21.81 ± 0.63	11.74* ± 0.91	22.16 ± 1.03	21 ± 0.5
liver GPx μmol (GSH) utilized/min/mg protein	9.03 ± 0.31	8.95 ± 051	6.56* ± 0.71	11.09 ± 0.92	10.03 ± 0.86
kidney GPx μmol (GSH) utilized/min/mg protein	6.99 ± 0.24	6.92 ± 0.45	4.8* ± 0.35	10.03* ± 0.64	8.66 ± 0.49

### Effect of NP on malondialdehyde (MDA) levels in plasma, liver, and kidney of experimental rats

NP exposure led to statistically significant increases in MDA levels across various tissues of the experimental rats (Table 4). In the plasma, MDA levels were 1.7 ± 0.02 nmol/ml for the control group, 4.23 ± 0.05 nmol/ml for the NP-exposed group (*P* < 0.05). In the liver, MDA levels were 1.03 ± 0.01 nmoles/mg tissue weight for the control group and 2.57 ± 0.02 nmoles/mg tissue weight for the NP-exposed group (*P* < 0.05). In the kidney, MDA levels were 0.57 ± 0.02 nmoles/mg tissue weight for the control group and 1.42 ± 0.04 nmoles/mg tissue weight for the NP-exposed group (*P* < 0.05). Results indicate a statistically significant increase in oxidative stress following nonylphenol exposure in all measured tissues.

**Table 4:** Effect of NP exposure on blood, liver and kidney MDA in rats treated with RES or NG

Parameter	NC	DMSO	NP	NP + RES	NP + NG
Plasma MDA (nmol/ml)	1.7 ± 0.02	1.72 ± 0.03	4.23* ± 0.05	1.52* ± 0.03	1.66 ± 0.04
liver MDA (nmoles/mg tissue protein)	1.03 ± 0.01	1.04 ± 0.02	2.57* ± 0.02	0.92* ± 0.01	1.01 ± 0.02
kidney MDA (nmoles/mg tissue protein)	0.57 ± 0.02	0.58 ± 0.01	1.42* ± 0.04	0.51 ± 0.03	0.56 ± 0.01

However, the co-administration of RES or NG effectively mitigated this effect, demonstrating MDA levels comparable to those of control and DMSO treated groups: 1.52 ± 0.03 and 1.66 ± 0.04 nmole/ml for NP + RES and NP + NG in the plasma respectively, 0.92 ± 0.01 and 1.01 ± 0.02 nmoles/mg tissue weight for NP + RES and NP + NG in the liver respectively, and 0.51 ± 0.03 and 0.56 ± 0.01 nmoles/mg tissue weight for DEP + RES and DEP + NG in the kidney tissue respectively. Results demonstrated statistically significant decreases in MDA level in plasma and liver in NP + RES group compared to the control and DMSO-treated groups, and no statistically significant differences in kidney MDA of NP + RES group and plasma, liver and kidney MDA of NP + NG group compared to the control and DMSO-treated groups (*P* < 0.05) as presented in Table 4.

### Impact of NP on glutathione-S-transferase (GST) levels in plasma, liver and kidney tissues

NP exposure induced statistically significant alterations in glutathione-S-transferase levels across plasma, liver, and kidney tissues of the experimental rats (Table 5). In the blood, NP exposure resulted in a significant increase in GST levels from 64.88 ± 1.38 U/dl in the control group to 78.37 ± 2.05 U/dl in the NP-treated group (*P* < 0.05). In the liver, GST levels reduced significantly from 6.18 ± 0.13 in the control group to 4.67 ± 0.2 in the NP treated group (*P* < 0.05). In the kidney, NP exposure led to a reduction in GST levels from 4.62 ± 0.1 in the control group to 3.28 ± 0.14 in the NP treated group (*P* < 0.05).

**Table 5:** Effect of NP exposure on blood, liver and kidney GST in rats treated with RES or NG

Parameter	NC	DMSO	NP	NP + RES	NP + NG
Blood GST (U/L)	64.88 ± 1.38	65.46 ± 1.94	78.37* ± 2.05	64.21 ± 1.11	66.32 ± 1.26
liver GST	6.18 ± 0.13	6.24 ± 0.18	4.67* ± 0.2	6.12 ± 0.1	6.32 ± 0.12
kidney GST	4.62 ± 0.1	4.66 ± 0.13	3.28* ± 0.14	4.97 ± 0.08	4.72 ± 0.09

Units of GST enzyme activity in liver and kidney are expresses as follows: µmol of CDNB–GSH conjugate formed/min/mg protein

However, administration of phytochemical compounds RES (RES) or NG (NG) effectively restored GST levels to those comparable with control and DMSO treated groups, with no statistically significant differences observed. Specifically, in the blood, GST levels in the NP + RES and NP + NG groups were 64.21 ± 1.11 U/dl and 66.32 ± 1.26 U/dl, respectively, while in the liver, GST levels were 6.12 ± 0.1 and 6.32 ± 0.12, respectively. In the kidney, GST levels were 4.97 ± 0.08 and 4.72 ± 0.09, respectively as shown in Table 5.

#### The impact of NP markers on endocrine and reproductive function

The impact of NP was pronounced, with statistically significant decreases in serum total testosterone levels compared to control groups (5.4 ± 0.64 ng/ml vs. 2.05 ± 0.17 ng/ml, (*P* < 0.05), concurrent increases in estradiol levels (33.26 ± 1.64 pg/ml vs. 51.22 ± 2.05 pg/ml, (*P* < 0.05), accompanied by a significant reduction in testis weight compared to both control and DMSO-treated groups function (3.43 ± 0.05 g vs. 2.51 ± 0.04 g, (*P* < 0.05). The recorded effects induced by NP were effectively countered by co-treatment with RES or NG, rendering the observed changes in testosterone levels, estradiol levels, and testis weight statistically non-significant compared to control and DMSO-treated groups (Table 6). Aside from testis weight, which remained significantly difference even after administration of NG (*P* < 0.05).

**Table 6:** Effect of NP exposure on estradiol, testosterone and testes weight in rats treated with RES or NG

Parameter	NC	DMSO	NP	NP + RES	NP + NG
Estradiol (pg/ml)	33.26 ± 1.64	32.79 ± 1.71	51.22* ± 2.05	35.22 ± 1.05	36.57 ± 1.36
Testosterone (ng/ml)	5.4 ± 0.64	5.23 ± 0.45	2.05* ± 0.17	4.67 ± 0.39	4.52 ± 0.9
Testes weight (g)	3.43 ± 0.05	3.4 ± 0.07	2.51* ± 0.04	3.27 ± 0.05	3.14* ± 0.08

### Interactions between ligands and estrogen receptors beta (ERβ), binding affinities and molecular interactions

Estradiol (E2) demonstrates a high affinity for estrogen receptor beta, with a binding affinity of -9.9 kcal/mole. This interaction involves several types of bonds, including hydrogen bonds with GLU 304, ARG 346, and HIS 475 residues, Pi-Pi T shaped bond with PHE 356 residue, and Alkyl and Pi-Alkyl bonds with multiple residues such as LEU 298 which is displaying two interactions, ALA 302, LEU 343, LEU 339, and LEU 476 as shown in Table 7.

**Table 7:** Illustrates the interaction between estrogen receptor beta and various ligands, including estradiol (E2), NP, Resveratrol, and Naringenin

Ligand	2D Interactions	Type of bonds	Interacted residues	Affinity (Kcal/mol)
Estradiol (E2)		Hydrogen bond	GLU 305 ARG 346 HIS 475	-9.9
		Pi-Pi T-shaped	PHE 356	
		Alkyl and Pi-Alkyl	LEU 298 (2) ALA 302 LEU 476 LEU 343 LEU 339	
Nonylphenol (NP)		Alkyl and Pi-Alkyl	LEU 339 LEU 298 LEU 476 (2) HIS 475 MET 295 ALA 302 (2)	-7.9
Resveratrol (RES)		Hydrogen bond	ARG 346 LEU 339 GLY 472 HIS 475	-7.9
		Pi-Sigma	LEU 339	
		Pi-Alkyl	LEU 343 ALA 302 LEU 476	
Naringenin (NG)		Hydrogen bond	VAL 338 ARG 346 GLU 305	
		Carbon hydrogen bond	GLU 305	-7.6
		Pi-Donor Hydrogen bond	VAL 280	
		Pi-Cation and Pi-Anion	ARG 346 GLU 305 LYS 401	
		Pi-Pi Stacked	HIS 279	
		Pi-Alkyl	PRO 277	

Regarding NP displays, it displays an affinity of -6.3 kcal/mole for estrogen receptor beta, interacting primarily through Alkyl and Pi-Alkyl bonds with residues such as LEU 339, LEU 298, HIS 475, MET 295, and LEU 476 and ALA 302, where both share two interactions as presented in Table 7.

RES exhibits an affinity of -7.9 kcal/mole with estrogen receptor beta, forming hydrogen bonds with ARG 346, LEU 339, GLY 472, and HIS 475 residues, Pi-Sigma bond with LEU 339 residue, and Pi-Alkyl bonds with residues including LEU 343, ALA 302, and LEU 476 as displayed in Table 7.

NG demonstrates a binding affinity of -7.6 kcal/mole with estrogen receptor beta, engaging in six different types of bonds. These include hydrogen bonds with VAL 338, ARG 346, and GLU 305 residues, Carbon hydrogen bond with GLU 305, Pi-donor hydrogen bond with VAL 280 residue, Pi-cation and Pi-anion bonds with residues such as ARG 346, GLU 305, and LYS 401, Pi-Pi stacked bond with HIS 279 residue, and Pi-alkyl bond with PRO 227 residue as reported in Table 7.

## Discussion

Our study investigated the toxic effects of NP on the antioxidant capacity, cellular and endocrine functions, underscoring the potential protective effects of phytochemical compounds such as RES and NG. Findings revealed significant alterations in various antioxidant key players in response to NP exposure, indicating a significant impact on systemic, liver, and kidney oxidative status. Namely, superoxide dismutase (SOD) is a vital antioxidant enzyme responsible for scavenging superoxide radicals while maintaining cellular redox balance. We reported a significant reduction in SOD activity in serum, liver, and kidney tissues following NP exposure. This decline in SOD levels suggests impaired antioxidant defense mechanisms. Catalase is a critical antioxidant enzyme involved in the breaking hydrogen peroxide to water and oxygen, thus protecting cells from oxidative damage. We recorded a significant decrease in catalase levels in serum, liver, and kidney tissues following NP exposure. Additionally, Glutathione peroxidase plays a key role in reducing hydrogen and lipid peroxides, thus protecting cells from oxidative damage. Our finding demonstrated significant reductions in GPx levels in blood, liver, and kidney tissues following NP exposure. Collectively, indicating compromised antioxidant defense mechanisms. However, co-administration of RES or NG effectively restored SOD, catalase and GPx levels, highlighting their protective effects against NP-induced oxidative stress.

Further, investigating glutathione-S-transferase, a marker involved in detoxification processes by catalyzing the conjugation of glutathione to various electrophilic compounds, we reported alterations in GST levels across plasma, liver, and kidney tissues following NP exposure, indicating disrupted detoxification mechanisms. On the contrary, we observed a significant increase in MDA levels in plasma, liver, and kidney tissues following NP exposure. Malondialdehyde is a marker of lipid peroxidation and oxidative stress. The co-administration of RES or NG effectively mitigated this effect, restoring MDA levels to near-baseline levels.

Several studies have reported similar findings regarding the detrimental effects of NP on antioxidant enzymes and markers. For instance, Kourouma et al. (2015) demonstrated that NP exposure led to a significant difference in SOD, CAT, MDA and GPx activity in liver tissues of rats exposed to NP compared to control group, consistent with our findings in both serum and tissue samples. Additionally, a significant decrease in catalase, superoxide dismutase and reduced glutathione levels in kidney tissues following NP exposure, supporting our findings on the impact of NP on antioxidant capacity in kidney tissue (Shi et al. 2021).

We explored the potential protective effects of RES and NG against NP-induced oxidative stress, consistent with previous research highlighting the antioxidant properties of these compounds. Although we do not find direct evidence of RES protective impact on tissue exposed to NP, the effect of RES was investigated with bisphenol A, a comparable xenoestrogen. RES supplementation attenuated oxidative stress and inflammation in a rat model treated with bisphenol A, corroborating our findings on the restoration of antioxidant enzyme levels by RES (Akash et al. 2023). Similarly, Khodayar et al. (2020) demonstrated that NG supplementation protected against bisphenol A induced toxicity in mice by enhancing antioxidant enzyme activities, supporting our observations on the protective effects of NG against NP-induced oxidative damage. Besides, the findings of our study underscore the significant impact of NP exposure on endocrine and reproductive function, as evidenced by pronounced alterations in serum hormone levels and testis weight. Specifically, we observed a substantial decrease in serum total testosterone levels, coupled with concurrent increases in estradiol levels, following NP exposure. These hormonal changes were accompanied by a significant reduction in testis weight compared to control and DMSO-treated groups.

Our study’s findings align with prior research documenting the endocrine-disrupting effects of NP exposure. Several studies investigated the effects of NP exposure on endocrine function in male rats and observed a significant decrease in serum testosterone levels and an increase in estradiol levels (Huang et al. 2019). These findings are consistent with our results, indicating that NP exposure induces hormonal imbalances favoring estrogenic activity over androgenic activity.

Furthermore, our study demonstrates the potential therapeutic efficacy of RES (RES) and NG (NG) in mitigating NP-induced endocrine and reproductive dysfunction. Co-treatment with RES or NG effectively normalized serum testosterone and estradiol levels, as well as testis weight, to levels comparable to control and DMSO-treated groups. These findings suggest that RES and NG may have protective effects against NP-induced endocrine disruption and reproductive toxicity, these findings are consistent with several previous studies (Thirumalaisamy et al. 2022; Juan et al. 2005; Bordbar et al. 2023; Alboghobeish et al. 2019). To gain deeper insights, we carried out molecular docking studies which revealed the interactions between NP, RES, NG, and estrogen receptor, providing a possible mechanism for their potential estrogenic activities. For instance, NP exhibited moderate binding affinity for estrogen receptor, while RES and NG demonstrated stronger binding affinities, suggesting their potential estrogenic activities. These interactions highlight the complexity of NP-induced endocrine disruption and point to the potential protective effects of RES and NG against NP-induced hormonal alterations.

### Toxicity mechanisms of NP

Exposure to NP increase ROS synthesis more specifically superoxide anions and H2O2 levels due to a robust increase in NOX4 (a ROS-generating NADPH oxidase) at both mRNA and protein levels and decrease the expression of phase 2 metabolizing enzymes (GST and UGT (uridine 5-diphospho-glucuronosyltransferase) which play an important role in the detoxification of exogenous substances, carcinogens and oxidative stress products. So that NP induce disruption of redox homeostasis and elevate oxidative stress. NP exposure reduced the expression of cytoplasmic and nuclear Nrf2 (nuclear factor E2- related factor) that responsible for the transcriptional activity of antioxidant enzymes, leading to increase in oxidative stress, cell apoptosis, and then decreasing cell viability as well as protein expression (Derakhshesh et al. 2017). As a result of high oxidative stress state, NP induce an increase in lipid peroxidation (MDA level), indicating heightened membrane damage. Importantly, immunoblot data revealed that exposure to NP in vivo induces a sharp increase in Erα (estrogen receptor alpha) immunoreactive protein and abrogates hepatic Erβ expression. Moreover, an increase in p38 MAPK (p38 Mitogen activated protein kinase) phosphorylation (activation) revealed elevated stress response and participation of ER subtypes in mediating the actions of NP in rats (Urmi et al. 2022).

The enzyme functioning in the production of testosterone, led to the reduction of serum testosterone in male rats exposed to NP (Han et al. 2004). Other mechanisms through which NP suppresses the levels of plasma testosterone could be inhibition or disturbances in the activity or function of steroidogenesis enzymes like 3β-HSD (3β-Hydroxysteroid dehydrogenase), 11β-Hydroxylase,17 α-Hydroxylase, Cyp11a1 (Cytochrome P450 Family 11 Subfamily A Member 1) and Star (Steroidogenic acute regulatory protein) which are involved in the synthesis of testosterone (Laurenzana et al. 2002; Labadie and Budzinski 2006; Ying et al. 2012). Decreased androgen levels along with increased 17β-Estradiol levels after exposure to NP are probably the consequences of increased aromatization of androgens (Soverchia et al. 2005). Elevation of plasma estradiol levels may be linked to the inhibition of glucuronidation and sulfation of estradiol by NP, glucuronidation and sulfation are process called conjugation in the liver plays an important role in the deactivation, excretion and clearance of active steroid hormones (Thibaut and Porte 2004; Yang et al. 2008).

### Potential protective mechanisms of RES and NG

RES and NG are considered as nuclear factor E2- related factor (NRF2) activators, whereas enhancing NRF2 activity increases the expression of antioxidant enzymes and the defense against oxidative stress. NRF2 was found to be a transcription activator of NADPH Quinone Dehydrogenase 1 (NQO1 gene) that bound to the antioxidant response element (ARE) in the promoter (Venugopal and Jaiswal 1996). NRF2–ARE signalling has a central role in the regulation of antioxidant gene expression. ARE, the cis element of NRF2 binding, is more accurately called the electrophile response element (EpRE) as the ‘antioxidant’ inducers are electrophiles and include hydrogen peroxide (H_2_O_2_), some components of intermediary metabolism and products derived from dietary polyphenols like RES and NG (Forman et al. 2014). NRF2–EpRE signaling regulates the basal and inducible expression of more than 200 genes that encode proteins involved in antioxidant defence, detoxification, apoptosis, DNA repair, removal of oxidized protein by the proteasome, inflammation and other processes (Ushida and Talalay 2013; Kobayashi 2016), deficiency of NRF2 signaling suppresses the induction of target antioxidant enzymes in response to oxidative stress, increases susceptibility to oxidative damage (Ma 2013) and accelerates the inflammatory response. In response to oxidative stimuli, KEAP1 (Kelch- like ECH- associated protein 1) is oxidatively modified and loses the capacity to present NRF2 for degradation. NRF2, both dissociated from KEAP1 and newly synthesized, escapes from degradation and is then translocated into the nucleus where it forms heterodimers with small Maf (musculoaponeurotic fibrosarcoma) or Jun family proteins (transcription factors), binds to EpRE in the promoter and increases transcription of target genes. RES could potentiate the activity of Nrf2/HO-1, resulting in a decline in the ROS content. It was also found that RES or NG activates the SIRT1/AMPK and rasing the antioxidant defenses by the Nrf2 pathway (Bhattarai et al. 2016). RES potentiates the expression of Nrf2 through dissociation of Nrf2-Keap1 binding and increases the translocation of Nrf2 into the nucleus. RES dissociates the bindings between Nrf2-Keap1 through increase the interaction between p62-Nrf2. RES also activates Nrf2/ARE via stimulation p38 MAPK and SIRT1/FOXO1 signaling. RES elevates the Nrf2 expression through suppression of the inhibitory signaling (Tahereh et al. 2020; Zhang et al. 2017; Wang et al. 2017; Lou et al. 2014).

Overall, our study contributes to a deeper understanding of the toxic effect of NP on antioxidant capacity and endocrine functions. Besides, it underscores the potential therapeutic utility of RES and NG in alleviating these adverse effects.

## Data Availability

The datasets used and/or analysed during the current study available from the corresponding author on reasonable request.
